# Based on Sportomics: Comparison of Physiological Status of Collegiate Sprinters in Different Pre-Competition Preparation Periods

**DOI:** 10.3390/metabo14100527

**Published:** 2024-09-29

**Authors:** Pengyu Fu, Xiaomin Duan, Yuting Zhang, Xiangya Dou, Lijing Gong

**Affiliations:** 1 Department of Physical Education, Northwestern Polytechnical University, Xi’an 710072, China; fupy@nwpu.edu.cn (P.F.);; 2 Shaanxi Institute of Sports Science, Xi’an 710065, China; hedyduan666@gmail.com; 3 College of Life Science, Northwestern Polytechnical University, Xi’an 710072, China; 4 Key Laboratory of Exercise and Physical Fitness, Ministry of Education, Beijing Sport University, Beijing 100084, China

**Keywords:** sprinters, collegiate athletes, pre-competition status, sports performance, gut microbiomes

## Abstract

This study aimed to assess the impact of pre-competition training by comparing the differences of collegiate sprinters in physiological state between strengthening and tapering training period by sportomics and combining their sport performance. Fifteen collegiate sprinters were investigated or tested on their body composition, dietary habits, energy expenditure, sleep efficiency, heart rate and respiratory rate during training, blood (blood cells, biochemical and immune markers) and urine (urinalysis), gut microbiome distribution, microbiome and blood metabolites, and their functions during the strengthening (Group A) and tapering training period (Group B) prior to competing in the national competitions. We found that 26.67% of sprinters achieved personal bests (PB) after the competition. The limb skeletal muscle mass and lymphocyte ratio of male sprinters in Group B were lower than those in Group A, and the serum creatine kinase (CK) level was higher than Group A (*p* < 0.05). The levels of serum CK, interleukin-6 (IL-6), interleukin-1β (IL-1β), and urine-specific gravity (SG) of the two groups were higher than the upper limit of the reference value. The detection rates of urine white blood cell (WBC) and protein in Group B were higher than those in Group A. The gut microbiome health index (GMHI) of Group A was higher than that of Group B, and the microbial dysbiosis index was lower than that of Group B. The ratio of *Firmicutes*/*Bacteroidota* (F/B) in Group A was higher than that in Group B. There were 65 differential metabolites in the A/B group, and the enriched pathway was mainly the NF-kappa B signaling pathway (up); B/T cell receptor signaling pathway (up); Th1 and Th2 cell differentiation (up); phenylalanine metabolism (up); and growth hormone synthesis, secretion, and action (up). There were significant differences in blood metabolites between the A and B groups, with a total of 89 differential metabolites, and the enriched pathway was mainly the NF-kappa B signaling pathway (up), T cell receptor signaling pathway (up), Th1 and Th2 cell differentiation (up), and glycerophospholipid metabolism (down). In conclusion, the imbalance of the gut microbiome and inflammation and immune-related metabolites of collegiate sprinters during the pre-competition tapering training period may be the cause of their limited sports performance.

## 1. Introduction

The effectiveness of pre-competition training programs is directly impacted by their scientificity and logic. Pre-competition training typically consists of strengthening and tapering the training period. Sprinting is a kind of speed-strength sport, and therefore training volume usually reaches peak three weeks prior to competition and then gradually drops [1]. Collegiate sprinters face pressure from both training and competition, as well as from academics and work, which may significantly differ from professional athletes’ psychological and physiological pre-competition conditions [2]. However, there are not many studies on this topic.

Monitoring athletes’ pre-competition training plays an important role in developing reasonable training programs. It is known to all that a variety of physiological and biochemical markers, such as body composition, heart rate, respiratory rate, and blood and urine indicators, are frequently used for sports training monitoring. The changes of these markers help coaches and athletes thoroughly understand the physical condition and training effect of athletes by reflecting the nutrition and metabolic health, hydration balance, muscle status, endurance performance, fatigue, and inflammation levels. Although these biomarkers have been widely used, the analysis of their results still faces many challenges: (1) the sensitivity of single biomarkers to detect overtraining is limited, (2) the reference range for different training levels has not been clearly defined, and (3) interindividual variance in absolute values and relative changes in biomarkers [3].

Therefore, systematically assessing the impacts of pre-competition training modifications based on single metabolites and fewer pathways is frequently challenging in traditional investigations. Metabolomics and other omics technologies are applied to investigate the metabolic effects of physical exercise on individuals, which is known as “sportomics”, having gained popularity in the field of exercise physiology [4,5]. We attempted to apply “sportomics” to assess, as is rarely documented, the effects of various pre-competition preparation phases on collegiate sprinters’ condition.

Collegiate athletes train at a lesser volume and intensity than professional athletes. Conventional physiological and biochemical markers might not be accurate predictors of pre-competition training conditions. Drawing on “sportomics”, metabolomics and microbiomics were used in our study. Changes in metabolic levels can be detected by metabolomics in real time, within hours, or even days [6], which have been used to identify metabolites at various competition and preparation nodes in competitive sports [7,8]. “Providing fuel for microbes” could develop into a key tactic for enhancing sports performance. This concept has made the microbiome a research hotspot in competitive sports [9]. The microbiome has been employed in some recent research to monitor athlete training, and studies show that this is a highly significant tool for understanding possible inflammatory hazards and creating individualized training regimens [10].

We hypothesized that gut microorganisms and their metabolites, as well as blood metabolites during the pre-competition strengthening and tapering training period, are different, which, combined with the variations in physiological and biochemical markers, can help predict sports performance. This study aims to evaluate the rationality of the pre-competition training program of collegiate sprinters by searching for more sensitive pre-competition training monitoring markers and provide theoretical guidance for the formulation of scientific and effective pre-competition preparation programs.

## 2. Materials and Methods

### 2.1. Participants and Groups

This study involved 15 collegiate sprinters, who were training at Northwestern Polytechnical University, at the period of preparation for the 21st National Collegiate Track and Field Championships of China in 2023. The sprinters were 21.20 ± 1.74 years old and had been accepted into the professional training for 6.8 ± 2.18 years. All were national second-level or above (sports technology level of China) athletes, and 10 of them were male and 5 were female. The sprinters shared a lifestyle (lived and ate together) and pre-competition training before the competition. Subjects were excluded if they (1) were unable to train because of a recent injury; (2) used antibiotics and probiotic supplements in the previous 6 months; and (3) had gastrointestinal diseases, such as diarrhea and constipation.

**Figure 1:**
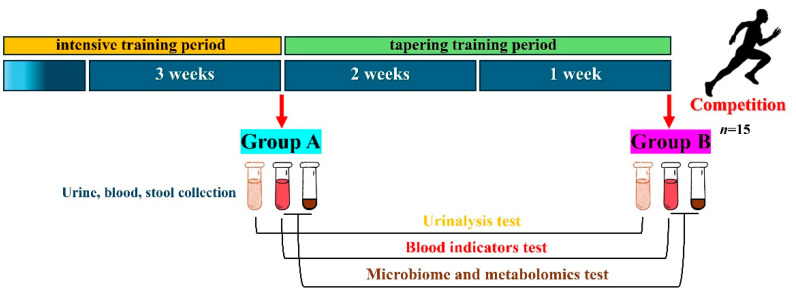
Conditions for collecting biological samples from sprinters.

We monitored the physiological conditions of the sprinters during the strengthening and the tapering training period and divided these two periods into Group A (*n* = 15) and Group B (*n* = 15) (Figure 1).

The training plans for two periods are shown in Table 1. All sprinters completed informed permission forms after fully understanding the research purpose and program with the coaches’ assistance and cooperation.

### 2.2. Body Composition

The direct digital detectors (GE PRODIGY) were used to test the body composition of sprinters in a fasting state.

### 2.3. Dietary Survey

The 24 h recall method (http://www.ihatepsm.com/blog/24-hour-recall-questionnaire-method, access date: 1 July 2023) was used to record the daily dietary intake (including food types and estimated weights of three meals and snacks) by the BooHee Health mobile ‌application (APP) (this APP can estimate the weight of food and calculate energy expenditure) one week before the competition [11].

### 2.4. Energy Expenditure and Sleep Efficiency

The three-axis accelerometers (ActiGraph wGT3X-BT, ActiGraph, Pensacola, FL, USA) were worn on the left wrist throughout the day to record daily physical activity energy expenditure, average activity intensity, and sleep efficiency.

### 2.5. The Test of Heart Rate and Respiratory Rate

The Zephyr physiological status monitoring training system was worn on the chest to record the heart rate and respiratory rate during strength training and special athletics training.

### 2.6. Blood and Urine Indicators

A total of 10 mL of fasting venous blood was drawn to test blood cells and biochemical and immunological indicators; 15 mL of the first mid-morning urine was collected, and routine urine tests were performed using a urine analyzer. The above tests were completed in the University Hospital.

### 2.7. Gut Microbiome Sequencing and Analysis

The middle section of the first stool in the morning was collected in a 2 mL collection tube and frozen with liquid nitrogen. The Illumina PE300/PE250 platform (Illumina, San Diego, CA, USA) was used for the high-throughput sequencing of the 16S rRNA V3-V4 region in stool samples. Bioinformatic analysis of the gut microbiome was carried out using the Majorbio Cloud platform (https://cloud.majorbio.com, access date: 1 September 2023). Bacterial community characterization index and composition were analyzed.

### 2.8. Metabolomics Sequencing and Analysis

A total of 50 mg of stool and 100 µL of urine samples were weighed and extracted with 400 µL of extraction solution, respectively. The liquid chromatography–mass spectrometry system (LC-MS/MS) analysis of samples was conducted on a Thermo UHPLC-Q Exactive HF-X system equipped with an ACQUITY HSS T3 column at Majorbio Bio-Pharm Technology Co., Ltd. (Shanghai, China). The pretreatment of LC/MS raw data was performed by Progenesis QI (Waters Corporation, Milford, CT, USA) software, and a three-dimensional data matrix in CSV format was exported. The data matrix obtained by searching the database was uploaded to the Majorbio cloud platform (https://cloud.majorbio.com, access date: 1 September 2023) for data analysis. The R package “ropls” (Version 1.6.2) was used to perform principal component analysis (PCA) and orthogonal least partial squares discriminant analysis (OPLS-DA). The metabolites with variable importance in the projection (VIP) > 1, *p* < 0.05, were determined as significantly different metabolites based on the VIP obtained by the OPLS-DA model and the *p*-value generated by Student’s *t* test. Differential metabolites among two groups were mapped into their biochemical pathways through metabolic enrichment and pathway analysis based on the Kyoto Encyclopedia of Genes and Genomes (KEGG) database (http://www.genome.jp/kegg/, access date: 1 October 2023).

### 2.9. Statistical Analysis

All the data were analyzed using SPSS20.0 and expressed as mean ± standard deviation (SD). The paired *t*-test was used to determine the statistical difference of body composition, energy metabolism, sleep efficiency, heart and respiratory rate, and blood and urine markers between the two groups. The gut microbiome characterization index between the two groups was analyzed using the Wilcoxon rank sum test (false discovery rate (FDR) correction). *p* < 0.05 was considered statistically significant (Cohen’s d reflects the effect size).

PCA was converted to unit variance (UV) with a confidence level of 0.95. Student’s *t*-test was used to compare the two groups in the differential metabolite analysis. The screening conditions were *p*-value < 0.05, VIP-pred-OPLS-DA > 1, and the fold change (FC) > 1. Seven-fold cross validation was used to ascertain the VIP using OPLS-DA/PLS-DA as the supervised model. KEGG pathway enrichment analysis was performed by relative-betweenness centrality and BH multiple test correction.

## 3. Results

### 3.1. Sports Performance and Body Composition

After the competition, four sprinters achieved their personal bests (PB), including three male and one female sprinter. There were no significant differences in body mass index (BMI), body fat percentage, and muscle mass between the two groups in male and female sprinters. The body fat percentage and trunk skeletal muscle mass of male sprinters in Group A increased compared with those in Group B, while the body fat percentage of female sprinters in Group A decreased, and the trunk skeletal muscle mass increased compared with those in Group B. The limb skeletal muscle mass of male sprinters in Group B was significantly lower than Group A (*p* < 0.01, Cohen’s d = 0.54), and which in female sprinters in Group B also decreased (Table 2).

### 3.2. Energy Metabolism and Sleep

**Figure 2:**
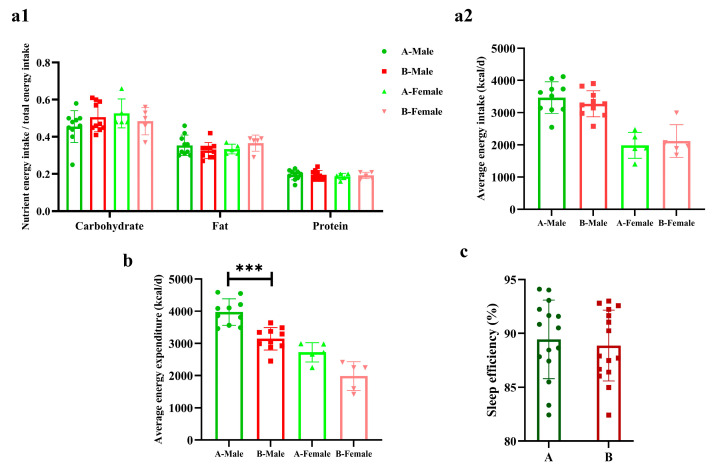
The energy metabolism and sleep efficiency of the sprinters (*n* = 15). (**a1**) The three major nutrient energy intake ratio; (**a2**) the average daily energy intake; (**b**) the average daily energy expenditure; (**c**) the sleep efficiency. Compared with Group A-Male, *** *p* < 0.001.

There were no differences in the three major nutrient energy supplies and energy intakes nor in sleep efficiency between the two groups. The energy expenditure of male sprinters in Group B was significantly lower than Group A (*p* < 0.001, Cohen’s d = 2.18), but there were no differences in females (Figure 2).

### 3.3. Heart Rate and Respiratory Rate during Training

The maximum heart rate (*p* < 0.05, Cohen’s d = 0.80) and mode of respiratory rate (*p* < 0.01, Cohen’s d = 1.04) in Group B were lower than those of Group A in strength training. The mode of heart rate in Group B was higher than that of Group A (*p* < 0.05, Cohen’s d = 0.89), while the mode of respiratory rate was lower than that of Group A (*p* < 0.01, Cohen’s d = 1.23) in special athletics training (Table 3).

### 3.4. Blood Markers

There was no significant difference in the levels of white blood cell (WBC), immunoglobulin A (IgA), red blood cell (RBC), ferritin, hemoglobin (HGB), mean corpuscular hemoglobin concentration (MCHC), testosterone, cortisol, testosterone/cortisol, blood urea nitrogen (BUN), lactate dehydrogenase (LDH), interleukin-6 (IL-6), or interleukin-1β (IL-1β) between the two groups. The lymphocyte ratio in Group B was lower than in Group A (*p* < 0.05, Cohen’s d = 0.99); the creatine kinase (CK) levels (M: *p* < 0.05, Cohen’s d = 1.44; F: *p* < 0.01, Cohen’s d = 2.71) in Group B were higher than in Group A. The levels of CK, IL-6, and IL-1β were higher than the reference value (Table 4).

### 3.5. Urine Markers

No glucose (GLU), blood (BLD), nitrite (NIT), urobilinogen (UBG), bilirubin (BIL), or ketone (KET) were detected in the urine of the two groups. The detection rates of urine WBC (reflecting inflammatory status) and protein (PRO) (reflecting training intensity) in Group B were higher than those in Group A. The pH values (reflecting dietary structure) of both groups were slightly acidic. The specific gravity (SG) (reflecting hydration status) levels were higher than the upper limit of the reference value (Table 5).

### 3.6. Gut Microbiome Characterization Index and Composition Analysis

**Figure 3:**
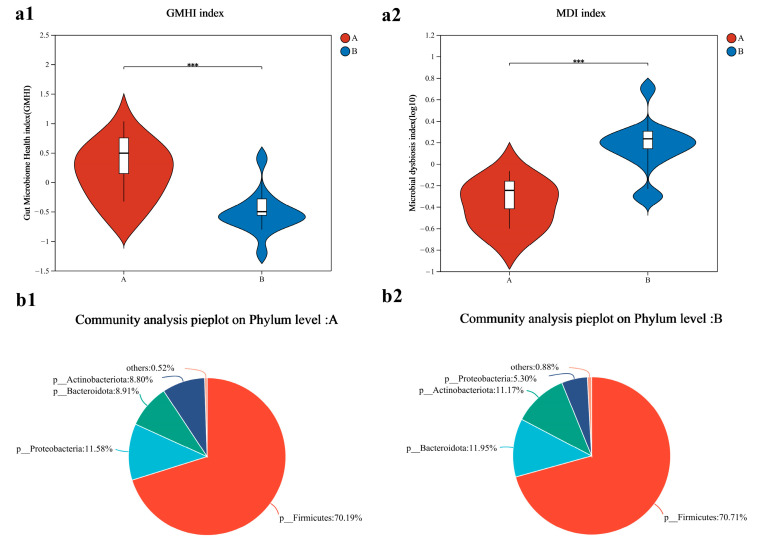
Bacterial community characterization index and composition analysis of sprinters (*n* = 15). (**a1**) The gut microbiome health index; (**a2**) the microbial dysbiosis index. Compared with Group A, *** *p* < 0.001. The community distribution of Group A (**b1**) and Group B (**b2**). “p” represents phylu.

The gut microbiome health index (GMHI) of Group A was higher than that of Group B (*p* < 0.001, Cohen’s d = 2.04) (Figure 3(a1)). The microbial dysbiosis index (MDI) in Group B was higher than that in Group A (*p* < 0.001, Cohen’s d = 2.26) (Figure 3(a2)). At the phylum level, the community composition of the two groups is shown in Figure 3(b1,b2), where the *Firmicutes*/*Bacteroidota* (F/B) ratio in Group A was higher than in Group B.

### 3.7. Metabolomic Analysis of the Gut Microbiome

**Figure 4:**
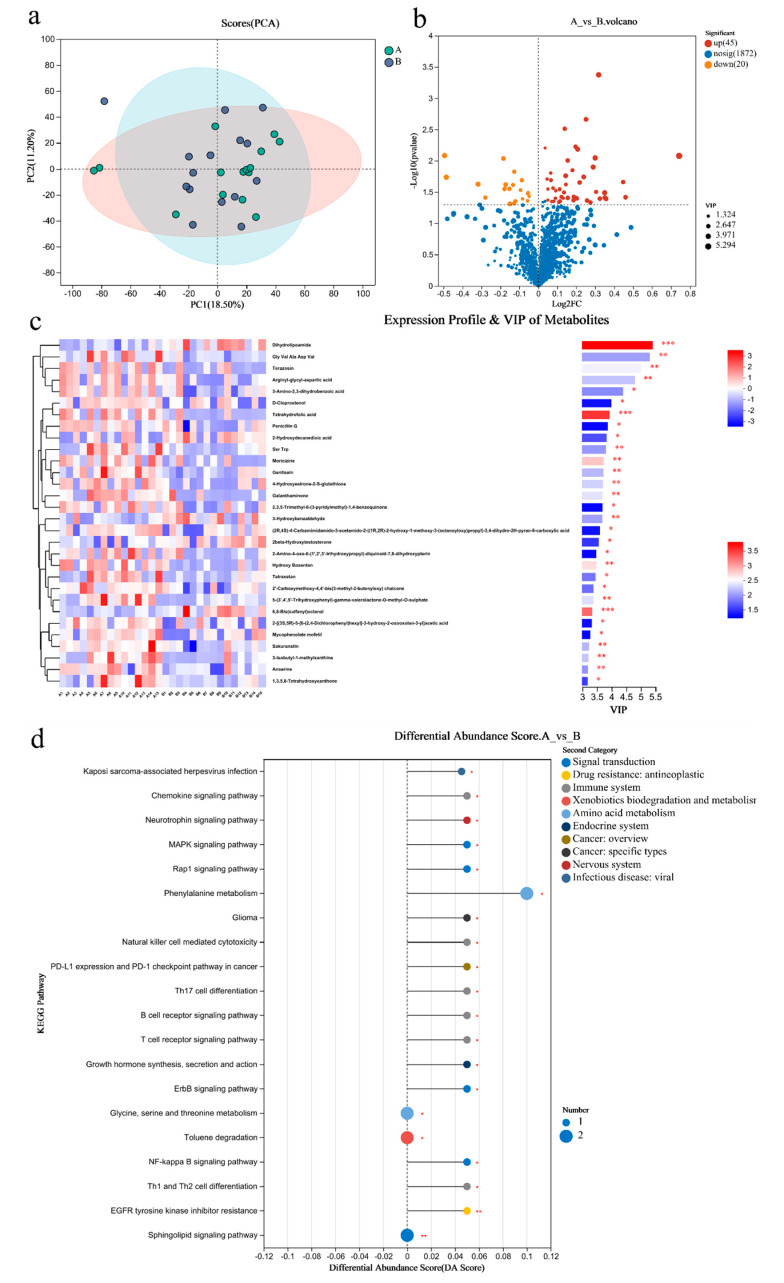
Differential metabolites of the gut microbiome of sprinters and their enriched pathways (*n* = 15). (**a**) The principal component analysis (PCA); (**b**) the volcano plot of differential metabolites; (**c**) the variable importance in the projection (VIP) analysis chart; (**d**) the differential abundance score plot of the KEGG pathway. Compared with Group A, * *p* < 0.05, ** *p* < 0.01, *** *p* < 0.001.

PCA analysis results showed that there was little difference in the metabolites of gut microbiome between the two groups (Figure 4a). There were 65 differential metabolites in Group A/B, of which 45 were upregulated and 20 were downregulated (Figure 4b). The top 30 differential metabolites with the highest VIP values are shown in Figure 4c, including anserine (up), etc. (Figure 4c). The pathways enriched by metabolites revealed that the inflammation and immunity related were NF-kappa B signaling pathway (up), B/T cell receptor signaling pathway (up), and Th1 and Th2 cell differentiation(up); amino acid metabolism was phenylalanine metabolism (up); endocrine system was growth hormone synthesis, secretion, and action (up) (Figure 4d).

### 3.8. Metabolomic Analysis of Blood

**Figure 5:**
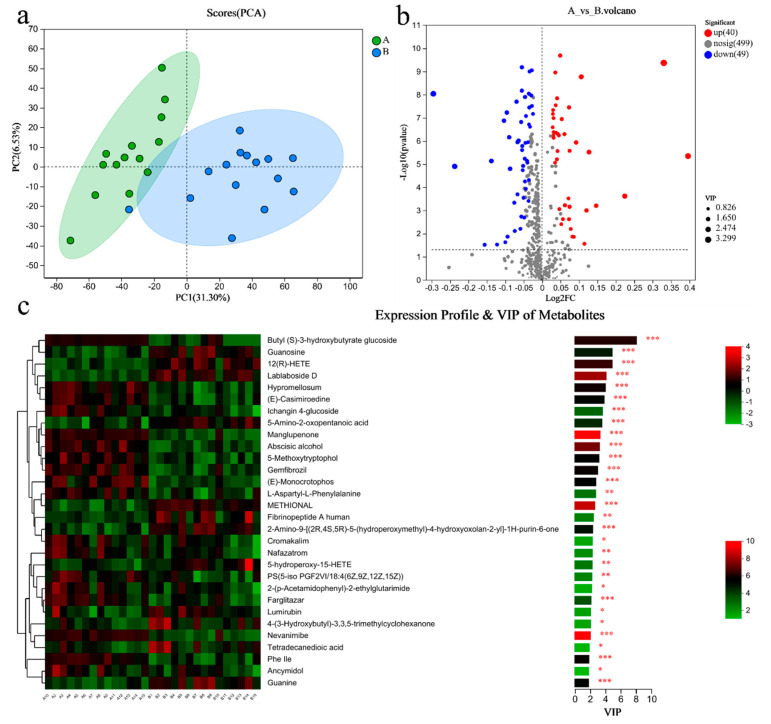

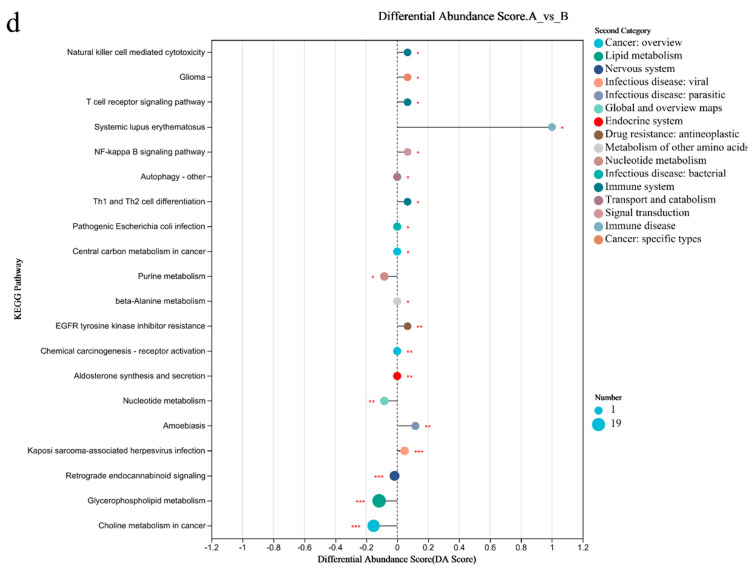
Differential metabolites of the blood of sprinters and their enriched pathways (*n* = 15). (**a**) The PCA. (**b**) The volcano plot of differential metabolites. (**c**) The VIP analysis chart. (**d**) The differential abundance score plot of the KEGG pathway. Compared with Group A, * *p* < 0.05, ** *p* < 0.01, *** *p* < 0.001.

PCA analysis results showed that there was a significant difference in the metabolites of blood between the two groups (Figure 5a). There were 89 differential metabolites in Group A/B, of which 40 were upregulated and 49 were downregulated (Figure 5b). The top 30 differential metabolites with the highest VIP values are shown in Figure 5c, including guanosine (down), guanine (down), etc. The pathways enriched by metabolites revealed that the related inflammation and immunity were NF-kappa B (NF-κB) signaling pathway (up), T cell receptor signaling pathway (up), Th1 and Th2 cell differentiation (up); the lipid metabolism was the glycerophospholipid metabolism (down) (Figure 5d).

## 4. Discussions

This study investigated the energy metabolism, training load, body composition, and blood and urine biochemical markers combined with the gut microbiome and metabolites of collegiate sprinters during the pre-competition strengthening and tapering training period to comprehensively analyze their pre-competition status and predict sports performance.

### 4.1. Diet, Training Load Adaptation, and Sports Performance

A healthy diet can help improve sports performance [12]. Sprints are characterized by their short duration, rapid pace, and high intensity. It is a typical sport that relies on anaerobic metabolism for energy supply and has high requirements on the speed and strength of athletes. In order to ensure adequate glycogen reserves and muscle mass, sprinters should increase their intake of carbohydrates and proteins before competitions. Pre-competition “overfilling” muscle glycogen is a crucial nutritional strategy for sprinters to enhance their sports performance [13]. A study of endurance runners showed that those who followed a high-carbohydrate diet had better sports performance than those following a high-protein diet [14,15]. Since energy expenditure and macronutrient composition are markers of dietary recommendations for altering body composition [16], we included these two variables to reflect the sprinters’ diet in this study. The recommended daily energy intake for males is 4080 kcal and for female is 3190 kcal, and the recommended carbohydrate daily intake is 663 g for males and 518 g for females, based on the weight of our sprinters [17]. We discovered that the sprinters’ overall energy intake and carbohydrate intake did not significantly increase between the two pre-competition periods, and the levels were lower than the recommended values: the mean daily energy intakes of male sprinters in Groups A and B were 3466 and 3275 kcal, respectively, while those of female sprinters were 1984 and 2120 kcal, respectively; the daily carbohydrate intakes of male sprinters were 394 and 413 g, respectively, while those of female sprinters were 261 and 251 g, respectively. This could have been one of the reasons for poor sport performance. We speculate that this may be related to the decreased appetite caused by sports fatigue, or it may be related to the fact that collegiate athletes have a relative lack of knowledge about sports nutrition and have not deliberately increased their energy and carbohydrate intake.

Furthermore, sprinters’ urine was sub acidic during both periods, and their SG was higher than the test value’s upper limit, suggesting that neither group consumed enough water [18].

In our study, the training volume was decreased throughout the tapering training period while the training load remained unchanged from the strengthening training period. Sprinters were less adaptive to training in special athletics training, as evidenced by a decrease in heart rate, which may be related to sports fatigue. This was also reflected in their urine biochemical markers, such as the higher detection rate of urine WBC and protein during the tapering training period. In contrast, sprinters were more adaptive during strength training.

### 4.2. Gut Microbiome and Sports Performance

The hypothalamic–pituitary–adrenal (HPA) axis can be activated by athletes undergoing high-intensity (over 60% of maximum oxygen consumption, VO_2max_) and long-duration (above 90 min) training prior to competition, as well as by the tension and anxiety that comes with it [19]. The gut–brain axis, which describes the reciprocal modulation between the gut and brain, is significantly impacted by the activity of the HPA axis. Certain bacterial populations may change as a result of this two-way communication, and the released metabolites may then alter host behavior [20].

We found that sprinters in the tapering training period had an unbalanced gut microbiota, a lower F/B ratio, and somewhat lower sleep efficiency. Because it is positively connected with the levels of short-chain fatty acids (SCFAs) [21] and VO_2max_ [22], the F/B ratio is thought to be a sign of the dynamic balance of the microbiome and is higher in elite athletes [23].

Sports fatigue could be attributed to microbiome imbalance of our sprinters. Combined with the results of blood indicators in this study, it can be found that there was a decline in the lymphocyte ratio of sprinters during the tapering training period. This is an important indication of immunosuppression (EIS). Studies have found that long-term training lasting over 90 min at a level of intensity higher than 65~75% VO_2max_ without enough recovery, might quickly cause exercise-induced EIS, which raises the risk of bacterial and viral infections of athletes [19,24]. Muscle microtrauma and gut microbiome changes caused by high-intensity exercise are both important mechanisms leading to EIS. The serum CK levels considerably increased during the tapering training period in our study, and it was found to be higher than the reference value during both periods. Lack of oxygen and muscle contraction during high-intensity exercise cause damage to the muscle cell membrane; increased permeability; and CK infiltration from the cells into the blood, leading to an increase in serum CK [25]. This leads us to the topic of the relationship between the gut microbiota and skeletal muscle. Through its impact on the gut–muscle axis, the gut microbiota eventually influences muscle function and facilitates the process of muscle synthesis from protein intake [26]. Thus, according to our study, abnormal dietary nutrient intake and microbiome imbalance may be linked to male sprinters’ decreased limb muscle mass during the tapering training period.

### 4.3. Metabolites and Sports Performance

Nowadays, sports science has made extensive use of metabolomics, a potent technique for precisely assessing metabolic pathways at the system level, to investigate metabolic responses that change depending on the type, intensity, and duration of exercise. While the majority of research has concerned individuals with metabolic disorders or those participating in amateur sports, some recent studies have concentrated on the use of metabolomics in professional athletes’ training monitoring [27]. It is commonly known that serum or plasma samples make excellent candidate samples for the discovery of biomarkers in metabolomics. In addition, a multitude of microbial metabolic processes found in feces lead to the synthesis or alteration of a broad spectrum of molecules, including vitamins, amino acids, and saturated fat fatty acids (SCFAs) as well as other bioactive molecules like polyphenols and secondary bile acids, which are vital for the regulation of physiological processes and are used to provide energy and necessary nutrients [28].

The pathways enriched by microorganisms and blood metabolites were primarily immunological and inflammatory pathways in our study. It was more evidence that the sprinters were fatigued, especially when the blood levels of IL-6 and IL-1β were higher than the upper limit of the permissible amount [29]. A study has shown that the NF-κB signaling pathway in skeletal muscle can be activated by the body’s hypoxic condition during intense exercise, putting the body in an oxidative stress state [30]. Similarly, our study revealed that the level of anserine in Group B of microbial metabolites was downregulated. It has been discovered that anserine can reverse the oxidative damage caused by exercise. Anserine supplements can increase the level of glutathione disulfide (GSSG) and the activity of superoxide dismutase (SOD), preserving the body’s antioxidant capability [31]. Furthermore, both microbial and blood metabolites were enriched in T cell immune regulation in this study. One of the most significant biological parameters linked to the risk of viral infection in highly physically active people appears to be the balance between T1 and T2 immune cells. For athletes to experience immunological modulation following intense exercise, T cells are essential. These include Th1 cells, which are gradually activated throughout the recovery phase after intense exercise, and T2 cells, whose IL-6 levels continue to rise and cause Th17 cells to differentiate, function as a pro-inflammatory factor, and eventually contribute to tissue inflammation [32].

In our study, sprinters’ pre-competition training also affected the levels of other metabolites, such as guanosine and guanine, which were upregulated in Group B. A study has shown that maximal physical exertion is accompanied by increased degradation of purine nucleotides in muscle, with the products of purine catabolism accumulating in plasma [33]. Glycerophospholipid metabolism was also downregulated in our study. A study found that the blood metabolite levels of multiple pathways related to glycerophospholipids were altered after the competition in collegiate soccer players [34]. A study has also shown that phospholipid-related metabolites such as phosphatidylcholine and phosphatidylserine play an important role in alleviating exercise fatigue [35].

## 5. Conclusions

Some collegiate sprinters had an unsuitable diet throughout the pre-competition tapering training period and were unable to adjust to the training load, which manifested as gut microbiota imbalance and increased expression of inflammatory and immune-related metabolites. We conclude that changes in microbial and metabolite levels may be a more sensitive way to monitor collegiate athletes training, because their training volume and load are lower than those of professional athletes and may not be sufficient to cause significant changes in the levels of traditional physiological and biochemical indicators. Further investigations into specific metabolic and microbiological indicators relevant to particular sports may be undertaken in the future.

## Figures and Tables

**Table 1:** Two-period training plan.

Times	Mon.	Tues.	Wed.	Thur.	Fri	Sat.
Strengthening Training Period (Group A)						
Warm-up exercise	Warm-up and run for 10 min, cool-down for 10 min					
Formal training	100 m run × 1 set + 60 m run × 2 sets + 30 m run × 2 sets	300 m run × 5 sets	Upper and lower limb and back strength training	200 m run × 4 sets + 150 m run × 4 sets	Hurdle jumping, hurdle running, hip and ankle mobility training	Full speed special athletics training
Cooling-down exercise	Stretching for 15 min					
Tapering training period (Group B)						
Warm-up exercise	Warm-up and run for 10 min, cool-down for 10 min					
Formal training	100 m run × 1 set + 60 m run × 1 set + 30 m run × 1 set	300 m run × 2 sets	Upper and lower limb and back strength training	-	-	-
Cooling-down exercise	Stretching for 15 min					

**Table 2:** Body composition of the sprinters (mean ± SD, *n* = 15).

Indicators (Unit)	Male (*n* = 10)		Female (*n* = 5)	
	Group A	Group B	Group A	Group B
Height (cm)	178.75 ± 3.19		169.10 ± 5.32	
Body weight (kg)	68.52 ± 5.25	68.24 ± 5.59	56.88 ± 3.49	56.25±3.32
Body mass index	21.37 ± 1.18	21.31 ± 1.25	19.92 ± 0.57	19.68 ± 0.54
Body fat percentage (%)	10.29 ± 3.64	11.51 ± 2.08	18.60 ± 2.17	17.22 ± 4.32
Skeletal muscle mass (kg)	32.81 ± 2.61	31.50 ± 2.22	24.18 ± 1.58	24.32 ± 1.11
Trunk skeletal muscle mass (kg)	6.68 ± 0.79	6.96 ± 1.05	6.40 ± 0.48	6.90 ± 0.78
Limb skeletal muscle mass (kg)	26.10 ± 2.10	25.04 ± 1.80 **	17.80 ± 1.11	17.42 ± 0.67

Compared with Group A, ** *p* < 0.01.

**Table 3:** Heart rate and respiratory rate during training of the sprinters (mean ± SD, *n* = 15).

Indicators (Times/min)			Group A	Group B
Strength training	Heart rate	Maximum	182 ± 29	164 ± 14 *
		Average	117 ± 17	113 ± 13
		Mode	115 ± 17	110 ± 13
	Respiratory rate	Maximum	423 ± 11	37 ± 6
		Average	23 ± 3	21 ± 2
		Mode	24 ± 5	20 ± 3 **
Specific athletics training	Heart rate	Maximum	185 ± 15	188 ± 20
		Average	120 ± 9	128 ± 9
		Mode	110 ± 19	124 ± 12 *
	Respiratory rate	Maximum	43 ± 5	46 ± 5
		Average	23 ± 7	24 ± 3
		Mode	24 ± 5	20 ± 3 **

Compared with Group A, * *p* < 0.05, ** *p* < 0.05.

**Table 4:** Blood indicators of sprinters (mean ± SD, *n* = 15).

Blood Indicators (Unit) (Reference Values)	Implication	Group A		Group B	
		Male (*n* = 10)	Female (*n* = 5)	Male (*n* = 10)	Female (*n* = 5)
WBC (10^9^/ L) (3.69~9.16)	Immune status	5.24 ± 0.89		5.35 ± 1.04	
Lymphocyte ratio (%) (24~48.4)		46.43 ± 6.85		39.20 ± 7.63 *	
IgA (g/L) (0.72~4.29)		2.24 ± 0.70		2.26 ± 0.73	
RBC (10^12/^L) (M: 4.3~5.8; F: 3.8~5.1)	Aerobic capacity	5.11 ± 0.39	4.52 ± 0.26	5.08 ± 0.39	4.64 ± 0.25
Ferritin (ng/mL) (M: 21.81~274.66; F: 4.63~204.00)	Aerobic capacity Nutritional status	101.02 ± 29.04	25.33 ± 12.68	123.30 ± 22.43	33.27 ± 16.59
HGB (g/L) (M: 130~175; F: 115~150)	Aerobic capacity Training loads	153.30 ± 8.29	129.20 ± 10.11	152.50 ± 8.72	132.60 ± 9.96
MCHC (g/L) (310~370)		350.10 ± 8.79	336.40 ± 12.10	355.00 ± 9.10	339.40 ± 10.78
Testosterone (ng/mL) (M: 2.49~8.36; F: 0.029~0.481)	Training loads Recovery ability	7.39 ± 1.32	0.48 ± 0.23	6.91 ± 1.56	0.52 ± 0.15
Cortisol (μg/dL) (4.26~24.85)		19.27 ± 4.09	17.66 ± 4.49	17.43 ± 3.35	17.47 ± 2.79
T/C		0.40 ± 0.09	0.03 ± 0.02	0.40 ± 0.10	0.03 ± 0.01
CK (U/L) (M: 38~174; F: 26~140)	Training intensity Muscle injury and recovery	191.76 ± 68.26	217.19 ± 53.62	286.65 ± 63.59 *	370.32 ± 59.07 **
BUN (mmol/L) (2.9~8.2)	Training volume	5.45 ± 1.08		5.11 ± 0.94	
LDH (U/L) (109~245)	Anaerobic capacity	163.23 ± 19.29		167.45 ± 25.49	
IL-6 (pg/mL) (≤7)	Inflammatory response	9.29 ± 5.29		12.87 ± 9.28	
IL-1β (pg/mL) (≤5)		17.28 ± 9.24		20.62 ± 13.20	

Compared with Group A, * *p* < 0.05, ** *p* < 0.01.

**Table 5:** Urine indicators of sprinters (mean ± SD, *n* = 15).

Urine Indicators		Group A	Group B
WBC	The detection rates	13.33%	35.71%
GLU		(-)	(-)
BLD		(-)	(-)
PRO		6.67%	35.71%
NIT		(-)	(-)
UBG		(-)	(-)
BIL		(-)	(-)
KET		(-)	(-)
pH (3~7.5)	The test value (Reference values)	5.63 ± 0.74	5.62 ± 0.74
SG (1.010~1.025)		1.028 ± 0.003	1.027 ± 0.003

(-) indicates not detected.

## Data Availability

Data are contained within the article.
